# Medication adherence in patients with nontuberculous mycobacterial disease

**DOI:** 10.1016/j.jctube.2025.100544

**Published:** 2025-06-18

**Authors:** Arthur Lemson, Fleur Dijkhuizen, Ralf Stemkens, Arjan van Laarhoven, Reinout van Crevel, Jakko van Ingen, Rob Aarnoutse, Wouter Hoefsloot

**Affiliations:** a Department of Pulmonary Diseases, Research Institute for Medical Innovation, Radboud University Medical Center, Nijmegen, the Netherlands; b Department of Pharmacy, Pharmacology and Toxicology, Research Institute for Medical Innovation, Radboud University Medical Center, Nijmegen, the Netherlands; c Department of Internal Medicine and Radboud Community for Infectious Diseases, Radboud University Medical Center, Nijmegen, the Netherlands; d Department of Medical Microbiology, Research Institute for Medical Innovation, Radboud University Medical Center, Nijmegen, the Netherlands

**Keywords:** Nontuberculous mycobacterial disease, *Mycobacterium avium* complex, Medication adherence, Proportion of days covered, 5-item medication adherence report scale, Beliefs about medicines questionnaire

## Abstract

**Objectives:**

We aimed to measure and understand medication adherence in patients with nontuberculous mycobacterial (NTM) disease.

**Methods:**

We enrolled adults with NTM disease receiving antimycobacterial treatment at Radboudumc, the Netherlands. Demographics and treatment outcomes were recorded, and medication adherence was assessed repeatedly. Adherence was measured using the Proportion of Days Covered (PDC) at 6 and 12 months, calculated from pharmacy records, and the 5-item Medication Adherence Report Scale (MARS-5). The Beliefs about Medicines Questionnaire (BMQ) explored reasons for (non)adherence.

**Results:**

Sixty-one participants were enrolled, with a mean age of 63.2 (±12.9) years. Forty-one (67 %) had pulmonary, and 20 (33 %) extrapulmonary NTM disease, with *M. avium* complex being the most prevalent species (n = 38; 62 %). One or more adverse effects occurred in 56/61 (92 %) participants. A favorable response was seen in 34/49 (69 %) and 25/29 (86 %) participants at 6 and 12 months, respectively. The median PDC was 100 % (IQR, 99–100 %) at both timepoints, and the MARS-5 remained 5 out of 5 throughout treatment, indicating high adherence. Participants’ beliefs about the necessity of NTM treatment, as assessed in the BMQ, consistently outweighed concerns.

**Conclusions:**

Medication adherence was high throughout the first year of NTM treatment, likely due to the perceived necessity of treatment and regular follow-up at our reference clinic.

## Highlights

• Medication adherence was high in a cohort with NTM disease from a Dutch national reference clinic.
• Adverse effects occurred in the majority of patients.
• Beliefs about the necessity of NTM treatment outweighed concerns.

## 1 Introduction

Nontuberculous mycobacterial (NTM) disease is caused by one of approximately two hundred species of environmental opportunistic mycobacteria. NTM pulmonary disease (PD) is the most prevalent disease manifestation, although extrapulmonary (EP) disease can also occur. The disease typically follows a slowly progressive course that often necessitates prolonged, multi-drug antibiotic treatment – usually lasting 12–24 months for NTM-PD and 6–12 months for EP-NTM [1]. Despite the lengthy treatment duration, outcomes often remain unsatisfactory [[2], [3], [4]], with improvements taking months to manifest. Moreover, treatment is often complicated by adverse effects and drug-drug interactions, requiring dose adjustments, temporary or permanent drug cessation, or switching to alternative drugs [5].

Medication adherence is frequently suboptimal among patients with chronic illnesses [[6], [7], [8]], yet it has not been appropriately studied in the context of NTM disease where treatment is particularly burdensome. A retrospective study from the Netherlands collected real-life prescription data from a national pharmacy dispensing database and calculated a 90 % adherence rate in patients with NTM-PD on a 3-drug treatment regimen; however, treatment was often discontinued prematurely [9]. Medication adherence can be measured through various methods, from direct observation of intake and electronic monitoring to more practical clinical strategies like calculations from pharmacy dispensing and refill records and self-reporting questionnaires. Combining assessment tools is recommended because it allows for an in-depth analysis of both intentional and unintentional nonadherence [10]. Furthermore, understanding the reasons behind (non)adherent behavior is equally important, but requires using interviews or disease-specific questionnaires.

This study aimed to measure and understand medication (non)adherence in a well-characterized, single-center cohort of patients with NTM disease by employing a combination of three different adherence assessment tools.

## 2 Patients and methods

### 2.1 Study design

We performed a prospective, observational, single-center study. Medication (non)adherence was measured using pharmacy (re-)fill records and the 5-item Medication Adherence Report Scale (MARS-5). To understand the reason behind medication (non)adherence, we employed the Believes about Medicines Questionnaire (BMQ). Assessments using both questionnaires were scheduled at 1, 2–4, 6 and/or 12 months on treatment. Both questionnaires were taken at least once and repeated if possible, depending on the duration of treatment at enrollment. Questionnaires were administered by a member of the study team during live clinical follow-up visits or were sent to participants for completion online.

### 2.2 Participants and study setting

This study was part of the Mycobacterial Cohort Study at Radboud university medical center (Radboudumc), Nijmegen, the national referral center for NTM disease in the Netherlands. Eligible participants were ≥18 years of age, diagnosed with either pulmonary or extrapulmonary NTM disease, and initiated on antimycobacterial treatment between 2022 and 2024 (not exceeding 12 months of treatment at the time of enrollment). All participants provided written informed consent. Approval was obtained from the ethical review board, METC Oost-Nederland (2021–13231; June 12, 2023).

At our center, patients with NTM-PD are treated by pulmonologists, while those with EP-NTM are managed by infectious disease specialists. Both populations receive thorough education and instructions regarding their treatment and are monitored by a multidisciplinary team, that includes a specialized nurse, during regular outpatient visits. Outpatient visits are typically scheduled at 1, 3, 6, 9, and 12 months post treatment initiation. Patients often initiate therapy during a scheduled hospital admission. After discharge, certain drugs for NTM disease are provided by the hospital’s outpatient pharmacy (e.g., clofazimine, imipenem-cilastatin) due to their limited availability in community pharmacies. Additionally, dosing of specific antibiotics (e.g., macrolides, aminoglycosides) is individualized based on therapeutic drug monitoring.

Demographics, medical history, baseline characteristics, follow-up data, and treatment outcomes were retrieved from the hospital’s medical records. Symptom severity prior to treatment initiation was classified as mild, moderate, or severe, based on clinical notes of the treating physician. Given that different outcomes are used to determine treatment response in NTM-PD and EP-NTM [1,11], available clinical, microbiological and/or radiographic outcomes from each participant were integrated to classify the response at 6 and 12 months as favorable or unfavorable.

### 2.3 Adherence measurements

#### 2.3.1 Pharmacy dispensing and refill records

Dispensing and refill records were obtained from the Radboudumc outpatient pharmacy, containing first fill and refill prescriptions, including dates, quantities, dosages, and intake frequencies of the dispensed medication. Adherence was measured by calculating the Proportion of Days Covered (PDC), based on (re-)fill data. Any drug modifications made collaboratively with the patient (e.g., permanent, or temporary stop, substitution, or dose or frequency adjustments) that resulted in an under- or oversupply of prescribed medication were retrieved from the medical records and adjusted for in the PDC calculation. Treatment modifications initiated solely by the patient were considered nonadherent behavior and therefore included in the PDC calculation.

The PDC was calculated for the treatment periods between 0–6 (PDC_0-6_) and 6–12 (PDC_6-12_) months for each oral antibiotic prescribed for ≥ 4 consecutive months, using the following formula:

$$\mathrm{PDC}=\left(\frac{\mathrm{Sum}\;\mathrm{of}\;\mathrm{days}\;\mathrm{covered}\;\mathrm{in}\;\mathrm{specified}\;\mathrm{time}\;\mathrm{frame}}{\mathrm{Number}\;\mathrm{of}\;\mathrm{days}\;\mathrm{in}\;\mathrm{specified}\;\mathrm{time}\;\mathrm{frame}}\right)\times 100$$

**Fig. 1:**
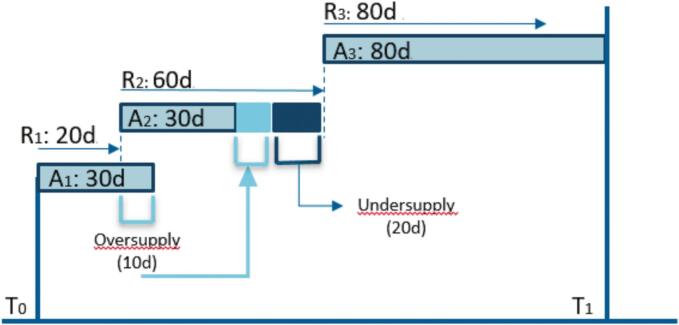
Fictional representation of a medication (re-)fill report to calculate the PDC. Abbreviations: T, Time; A, Drug ‘A’; d, Days; R, Refill. Drug A is prescribed for 20 days at T_0__(R_1_)_, but the first fill is dispensed for 30 days. The first refill (R_2_) is dispensed after 20 days, resulting in a 10-day oversupply. R_2_ is prescribed for 60 days but dispensed for 30 days. Even though the previous 10-day oversupply belonging to R_1_ is used to compensate for the shortage, a 20-day undersupply remains until the second refill (R_3_) is dispensed. The PDC for drug A between T_0_ and T_1_: $\mathrm{PDC}\;\mathrm{Drug}\;A=\left(\frac{140\;days}{160\;days}\right)\times 100=87.5\%$. []

Adapted from Arnet et al. 2013 29.

The mean PDC across all oral drugs in the regimen was determined by adding up the PDC for each drug and dividing the sum by the number of drugs. Fig. 1 visualizes the PDC calculation by illustrating a fictional (re-)fill record. A minimum of three (re)fills should be available for the PDC calculation to reflect a medication-taking behavior pattern and to represent a meaningful measure of adherence [12]. Additional study-specific criteria for PDC calculation are detailed in Table 1.

**Table 1:** Study-specific PDC-calculation criteria.

1. The PDC is calculated for oral antibiotics only
2. The PDC is calculated for antibiotics used ≥ 4 months with ≥ 3 prescriptions (re)filled
3. Macrolides (i.e., azithromycin and clarithromycin) are considered one drug when interchanged
4. Hospitalization days are not included in the specified time frame
5. Any undersupply directly after hospitalization is considered a ‘permissible gap’
6. Other permissible gaps are identified in the medical record and corrected for in the PDC calculation

Abbreviations: PDC, Proportion of Days Covered.

#### 2.3.2 5-item medication adherence Report scale (MARS-5)

The MARS-5 questionnaire measures self-reported medication adherence and has been validated in chronic illnesses such as asthma and hypertension [13,14]. It consists of 5 statements regarding medication use that should each be graded on a 5-point Likert scale (always = 1, often = 2, sometimes = 3, rarely = 4, never = 5), where a higher score indicates self-reported adherent behavior. The questionnaire includes one statement on unintentional nonadherence (“*I forget to take them*”) and four on intentional nonadherence (e.g., “*I decide to alter the dose*”). The final score is calculated by summing the scores of each item and by dividing the sum by the number of items, resulting in a range from 1 to 5. The copyright holder provided a Dutch version of the questionnaire. Participants were instructed to complete the questionnaire with respect to their overall drug regimen for NTM disease.

#### 2.3.3 Believes about Medicines questionnaire (BMQ)

The BMQ was developed to better understand patients’ perspectives on taking medication [15]. The questionnaire has been validated across various chronic conditions (e.g., asthma and diabetes mellitus) in both English and non-English speaking countries. It contains two domains: a general medication domain with 8 items and a disease-specific domain with 11 items, both answered on a five-point Likert scale (strongly disagree = 1, disagree = 2, uncertain = 3, agree = 4, strongly agree = 5). The final score of each domain is calculated by summing the scores and dividing the sum by the number of items, resulting in a range from 1 to 5. The general medication domain includes two subscales: harm, reflecting beliefs about the consequences of taking medication, and overuse, reflecting trust in the doctors’ decision to prescribe them. A high score (i.e., > 3; the scale’s midpoint) on the harm subscale indicates low confidence in a positive effect of medication in general. A high score on the overuse subscale indicates a low level of trust in the doctor’s decision to prescribe medication. The disease-specific domain contains a necessity and concern subscale that reflect the expected or perceived benefit of medication (for NTM disease) and concerns about having to use them, respectively. A high score on both subscales indicates either a strong belief in the necessity or greater concern. Additionally, a necessity-concern differential can be calculated by subtracting the concern score from the necessity score; a differential > 0 suggests beliefs about the necessity of NTM treatment outweigh concerns. Participants were instructed to complete the disease-specific domain in relation to their NTM treatment regimen.

### 2.4 Data analysis and statistics

Analysis was carried out using IBM SPSS Statistics version 29. Categorical data were presented as proportions, while continuous data were reported as mean (±SD) or median (IQR, 25th-75th percentile), depending on data distribution. In accordance with the BMQ scoring instructions, internal validity was checked for the BMQ general domain by determining Cronbach’s alpha. A 3-item overuse and 5-item harm (as opposed to 4-item overuse and 4-item harm) composition yielded the highest internal validity and was used in further analysis.

## 3 Results

### 3.1 Population characteristics

Among the 61 enrolled participants, 38 (62.3 %) were female and the mean (±SD) age was 63.2 (±12.9) years. Forty-one (67.2 %) participants were diagnosed with NTM-PD, including 14 (34.1 %) with nodular-bronchiectatic disease, 11 (26.8 %) with cavitary disease, and 16 (39 %) with a mixed form. Twenty (32.8 %) participants had EP-NTM, 15 (75 %) of which had a skin and/or soft tissue infection. *M. avium* complex (MAC) was the most prevalent causative species (n = 38, 62.3 %). Comorbidities were prevalent, for which 5.1 (±SD 3.8) concomitant drugs were taken (Table 2).

**Table 2:** Sociodemographic and clinical characteristics of 61 participants with NTM disease.

Characteristic	Value
**Age (years), mean ± SD**	63.2 (±12.9)
**Sex, female (%)**	38 (62.3 %)
**BMI (kg/m^2^), mean ± SD**	22.5 (±4.9)

**Country of origin**	
The Netherlands	58 (95.1 %)
Other	3 (4.8 %)

Education level*	
Low	23 (45.1 %)
Intermediate	15 (29.4 %)
High	13 (25.5 %)

**Employment status**	
Employed	20 (32.8 %)
Voluntary work	6 (9.8 %)
Unemployed	8 (13.1 %)
Retired	27 (44.3 %)

**Living status**	
Alone	17 (28.3 %)
Together	43 (71.7 %)

**Smoking status**	
Non-smoker	16 (29.6 %)
Ex-smoker	29 (53.7 %)
Current smoker	9 (16.7 %)

**Co-medication**	
Use of oral co-medication (yes, %)	56 (91.8 %)
Number of oral co-medications, mean ± SD	5.1 (±3.8)

**NTM disease manifestation**	
Pulmonary	41 (67.2 %)
Extrapulmonary	20 (32.8 %)

**NTM species**	
MAC	38 (62.3 %)
MAB	6 (9.8 %)
*M. chelonae*	4 (6.6 %)
*M. kansasii*	3 (4.9 %)
*M. marinum*	3 (4.9 %)
*M. xenopi*	1 (1.6 %)
*M. simiae*	1 (1.6 %)
*M. genavense*	1 (1.6 %)
*M. kyorinense*	1 (1.6 %)
*M. shimoidei*	1 (1.6 %)

**Symptom severity**	
Mild	17 (27.9 %)
Moderate	22 (36.1 %)
Severe	21 (34.4 %)

* In accordance with the central agency for statistics in the Netherlands. Values are expressed as N (%), unless indicated otherwise. Abbreviations: SD, standard deviation; BMI, body mass index; kg, kilogram; m, meter; NTM, nontuberculous mycobacteria; MAC, *mycobacterium avium* complex; MAB, *Mycobacterium abscessus*.

### 3.2 Antimycobacterial treatment

The majority of participants initiated NTM treatment at Radboudumc, 36 (59 %) during hospital admission and 20 (32.8 %) at the outpatient department. Two (3.3 %) participants began treatment during hospitalisation at other hospitals in the Netherlands, and 3 (4.9 %) started at outpatient departments elsewhere. Treatment adjustments were common, with 53 (86.9 %) participants experiencing one or more modifications (Table 3).

**Table 3:** Antimycobacterial treatment and adverse drug reactions.

Treatment	Value (N, %)
Oral antibiotics used*	
Azithromycin	56 (91.8 %)
Ethambutol	51 (83.6 %)
Clofazimine	44 (72.1 %)
Rifampicin	27 (44.3 %)
Clarithromycin	10 (16.4 %)
Isoniazid	3 (4.9 %)
Minocycline	2 (3.3)
Moxifloxacin	1 (1.6 %)
Levofloxacin	1 (1.6 %)

**Adverse effect present**	56 (91.7 %)
Ear and labyrinth	35 (57.4 %)
Gastro-intestinal	32 (52.5 %)
Skin-related	29 (47.5 %)
General malaise	26 (42.6 %)
Cardiac	17 (27.9 %)
Renal	11 (18 %)
Ocular	8 (13.1 %)
Hepatobiliary	8 (13.1 %)
Respiratory	8 (13.1 %)
Musculoskeletal	7 (11.5 %)
Neurological	4 (6.6 %)
Systemic	4 (6.6 %)
Infection	3 (4.9 %)
Blood and lymphatic	2 (3.3 %)
Other	15 (24.6 %)

**Drug modification**	
Premature permanent discontinuation	39 (63.9 %)
Temporary discontinuation	25 (41.0 %)
Substitution	13 (21.3 %)
Dose adjustment	50 (82.0 %)
Frequency adjustment	16 (26.2 %)

**Reason for drug modification**	
Adverse effect	38 (62.3 %)
Drug-drug interaction	11 (18.0 %)
Therapeutic drug monitoring	26 (42.6 %)
Insufficient effect	4 (6.6 %)
Standard dose reduction after loading phase**	38 (62.3 %)
Other	6 (9.8 %)

* The following parenteral and inhaled antibiotics were also prescribed but not included in the table: amikacin, imipenem-cilastatin, tigecycline, and amikacin liposomal inhalation and tobramycin.

** Refers to clofazimine. Abbreviation: SD, standard deviation.

Forty-two out of 61 (68.9 %) participants completed 6 months of NTM treatment; 12 (19.7 %) had not yet completed this duration, 5 (8.2 %) discontinued prematurely, and 2 (3.3 %) died during the first 6 months. Similarly, 25 (41 %) participants completed 12 months of NTM treatment; 9 (14.8 %) stopped prematurely, and 3 (4.9 %) died between 6 to 12 months of treatment. A favourable outcome was observed in 34 out of 49 (69 %) at 6 months and in 25 out of 29 (86 %) at 12 months of treatment.

### 3.3 Medication adherence

Pharmacy dispensing and refill records were available for 42 participants at 6 months and 23 participants at 12 months on treatment. The median Proportion of Days Covered (PDC) was calculated for 2 or 3 oral antibiotics per participant for those who fulfilled the study-specific criteria (Table 1) and found to be 100 % (IQR 98.8–100) during months 0 to 6 and 100 % (IQR 98.7–100) during months 6 to 12 of treatment.

Fifty-five (90.2 %) participants completed the MARS-5 and BMQ on one or more occasions (1 time: n = 24; 2 times: n = 15; 3 times: n = 14; 4 times: n = 2). All self-reported adherence outcomes are detailed in Table 4. The distribution of MARS-5 scores was heavily right-skewed, with a median score of 5 out of 5 throughout the first 12 months of treatment. The total MARS-5 score was therefore dichotomised into perfect adherence (MARS-5 = 5.0) and non-perfect adherence (MARS-5 < 5). At 1, 2–4, 6 and 12 months on treatment, the proportions of participants self-reporting perfect adherence were 80.6 %, 83.9 % 83.3 % and 73.3 %, respectively. The slight decrease in perfect adherence at 12 months was caused by a small increase in unintentional nonadherence (see supplement A).

**Table 4:** A cross-sectional presentation of BMQ and MARS-5 outcomes.

Adherence questionnaires	1 Month	2–4 Months	6 Months	12 Months
	(n = 32)	(n = 31)	(n = 26)	(n = 15)
**MARS-5**				
Median	5.0 (5.0)	5.0 (5.0)	5.0 (5.0)	5.0 (4.8–5.0)
Proportion with perfect adherence	25 (80.6 %)	26 (83.9 %)	23 (88.5 %)	11 (73.3 %)

**BMQ General**				
Overuse	2.2 (2.0–3.0)	2.0 (2.0–3.0)	2.2 (2.0–2.7)	2.7 (2.0–2.7)
Harm	2.2 (1.7–2.8)	2.2 (2.0–2.8)	2.1 (1.8–2.4)	2.0 (2.2–2.8)

**BMQ specific**				
Necessity	3.7 (2.8–4.0)	3.6 (2.8–4.0)	3.8 (3.0–4.0)	3.8 (3.4–4.2)
Concern	2.7 (2.3–3.0)	2.7 (2.2–3.2)	2.8 (2.2–3.2)	3.0 (2.2–3.5)
Differential	0.87 (0.3–1.4)	0.80 (−0.03–1.6)	0.87 (0.37–1.4)	0.47 (0.27–1.2)

Values are expressed as median (IQR) or N (%). Abbreviations: n, number; MARS-5, 5-item Medication Adherence Report Scale; BMQ, Believes about Medicines Questionnaire.

All four BMQ subscales and the necessity-concern differential remained numerically stable throughout the first 12 months of NTM treatment (see Table 4). Subscale scores for Harm and Overuse consistently fell below the midpoint of the scale, indicating above-average confidence in medication and doctors. Patient beliefs on NTM treatment necessity consistently outweighed the concerns, as reflected by the positive differential score at each timepoint. Responses to individual MARS-5 and BMQ items at all four timepoints are included in the supplements.

Associations between adherence outcomes, population characteristics, and treatment outcomes were not explored due to the small proportion of nonadherent participants.

## 4 Discussion

This study is the first to investigate medication adherence in patients with NTM disease using multiple adherence assessment tools. Despite including a frail population, characterized by advanced age, polypharmacy, and low educational level, treated with multidrug antibiotic regimens that were complicated by frequent adverse effects, medication adherence remained consistently high throughout the first year of treatment. This high adherence is likely attributable to the participants’ strong beliefs in the necessity of NTM disease treatment and the frequent, multidisciplinary follow-up at our reference clinic.

Medication adherence was measured using pharmacy (re-)fill records and the MARS-5. The calculated median Proportion of Days Covered (PDC) was 100 % for both the 0–6- and 6–12-month periods. The calculation included two to three oral antibiotics per participant, commonly a combination of a macrolide, ethambutol, clofazimine and/or rifampicin. In line with the calculated PDC adherence, self-reported adherence, as indicated by the MARS-5, was 100 % in approximately 80 % of patients throughout the first year on treatment.

Demographics, patient characteristics and the BMQ subscale scores were analysed to understand reasons for (non)adherent behaviour. The persistent belief in the necessity of NTM treatment- which consistently outweighed the concerns- alongside general confidence in medication and doctors reported on the BMQ, supports the observed adherent behaviour [16,17]. The positive necessity-concern differential is noteworthy, especially given the high number of recorded (92 %) and self-reported (approximately 54 % of participants) adverse effects, subsequent drug modifications (62.3 %) and suboptimal outcomes. Notably, most participants (n = 56, 92 %) initiated treatment at the national reference clinic for NTM disease and were closely monitored by a multidisciplinary team, which likely contributed to the observed adherence and strong beliefs in treatment efficacy.

Our study substantiates a previous study within the Netherlands that found a 90 % adherence rate for NTM-PD patients on a three-drug regimen, based on the Medication Possession Rate (MPR) calculated from pharmacy (re-)fill records [9]. This study however, included a short average treatment duration (120 days), relied on MPR rather than PDC, lacked corrections for intentional treatment modifications, and did not include a second adherence measure.

In comparison to our findings, adherence rates measured with similar tools in comparable chronic diseases, such as COPD, asthma, bronchiectasis, and chronic infections (e.g., tuberculosis, HIV), have consistently been lower [6,7,8,18,19,20,21,[22], [23], [24]]. For instance, in a one-year bronchiectasis study 45 out of 74 (61 %) participants self-reported adherent behaviour and a MPR ≥ 80 % was present in 39 out of 73 (53 %) for respiratory medicine including azithromycin [6]. Moreover, a 6-month PDC calculated based on pharmacy records in a South-Korean cohort with tuberculosis was 69 % (SD ± 33 %) [22]. Unfortunately, these studies did not utilize an adherence tool like the BMQ, which would provide insights into the reasons behind (non)adherence.

While associations between nonadherence and suboptimal treatment outcomes are frequently noted [6,7,8,25,26], the clinically meaningful adherence cut-offs, if known, differ across drug classes, chronic conditions, and population characteristics [27,28]. Currently, no adherence cut-off exists for NTM disease, and establishing one was not the focus of this study. Furthermore, due to the low number of nonadherent participants, we did not examine associations between adherence and outcomes. Nevertheless, despite the high level of adherence in this study, we observed a treatment response at 6 months (69 % favourable) that overlaps with previous studies, illustrating the challenge of managing NTM disease [1].

The adherence assessment tools employed in this study were selected to create a complementary approach. We opted for PDC over its predecessor, MPR, because it allows for correction of under- and oversupply of medication [29,30], which is particularly relevant in patients experiencing frequent drug modifications. Where possible, both PDC_0-6M_ and PDC_6-12M_ were calculated separately to correct for early and late adverse effects as well as perceived benefit of treatment, which typically occurs within 6 months. Downsides of the PDC are the fact that it remains an indirect adherence measure since it does not confirm actual medication intake and is a labour-intensive calculation that requires access to both pharmacy and medical records. The MARS-5 was included because of its practicality in longitudinal study design, capacity to differentiate between intentional and unintentional adherence, and detailed insights into intentional nonadherent behaviour (e.g., altering the dose or temporarily stopping). Finally, the BMQ provided valuable context regarding the beliefs of the studied population, that supported our primary finding. Previous research indicates that the BMQ can predict nonadherence [16,17] and may facilitate targeted intervention.

This study has four important limitations. First, its single-center design at a national reference center, where a multidisciplinary team closely and regularly monitors patients, likely influences treatment behaviour and perceptions. Extrapolation of results to other cohorts should therefore be approached with caution, as adherence outside a multidisciplinary setting may still be problematic. Additionally, participation in a prospective adherence study may alter medication-taking behaviour due to awareness of being observed, introducing a potential social desirability bias – a phenomenon known as the ‘Hawthorne effect’ [31]. Third, 24 participants completed adherence questionnaires once, while 31 had repeated assessments, which may have introduced a memory effect; however, a minimum 1-month interval was maintained between questionnaire administration. Lastly, our study population included both NTM-PD and EP-NTM patients who may exhibit different characteristics and total treatment durations, potentially affecting adherence behaviours and beliefs.

In conclusion, medication adherence was high throughout the first year of treatment for NTM disease in a national reference center. Participants’ beliefs in the necessity of antibiotic therapy consistently outweighed their concerns, despite frequent reports of treatment complications. We recommend the implementation of different assessment tools in future studies to gain comprehensive insight in (non)adherent behaviour in NTM disease.

## CRediT authorship contribution statement

**Arthur Lemson:** Writing – original draft, Investigation, Formal analysis, Data curation, Conceptualization. **Fleur Dijkhuizen:** Writing – review & editing, Investigation, Formal analysis, Conceptualization. **Ralf Stemkens:** Writing – review & editing, Conceptualization. **Arjan van Laarhoven:** Writing – review & editing, Investigation, Conceptualization. **Reinout van Crevel:** Writing – review & editing, Investigation, Conceptualization. **Jakko van Ingen:** Writing – review & editing, Conceptualization. **Rob Aarnoutse:** Writing – review & editing, Supervision, Conceptualization. **Wouter Hoefsloot:** Writing – review & editing, Supervision, Investigation, Conceptualization.

## Declaration of competing interest

The authors declare that they have no known competing financial interests or personal relationships that could have appeared to influence the work reported in this paper.
