# Supplementary material for: Medication adherence in patients with nontuberculous mycobacterial disease
Source: J Clin Tuberc Other Mycobact Dis. 2025 Jun 18;40:100544. doi: 10.1016/j.jctube.2025.100544 (PMC12221756; doi:10.1016/j.jctube.2025.100544)
Supplement: Supplementary Data 1 [file mmc1.docx]

**Supplementary material**

Overview of content

Results:

Figure S1. Responses to individual MARS-5 statements per study visit

Figure S2a-d. Responses to individual BMQ statements per study visit

Figure S1. Responses to individual MARS-5 statements per study visit


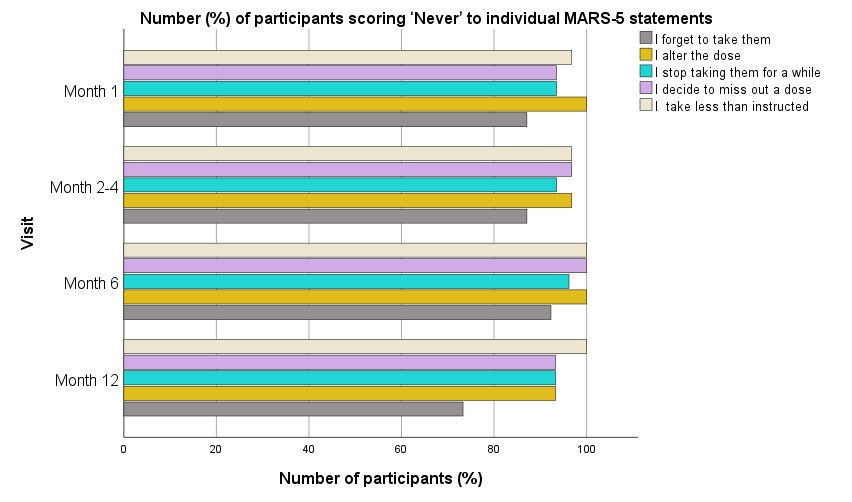


Supplementary S2a-d. Responses to individual BMQ statements per study visit

S2a.


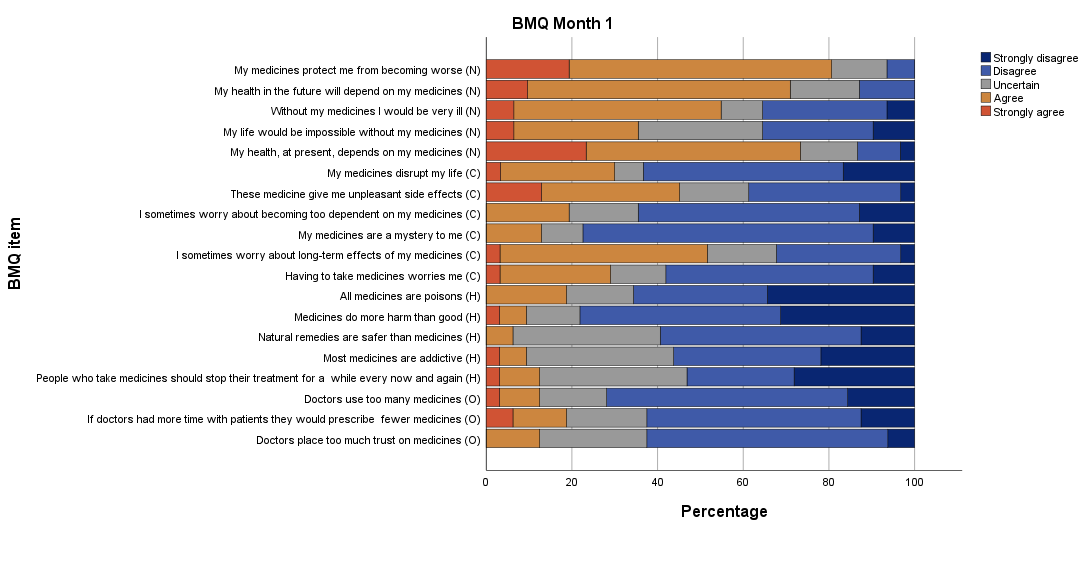


S2b.


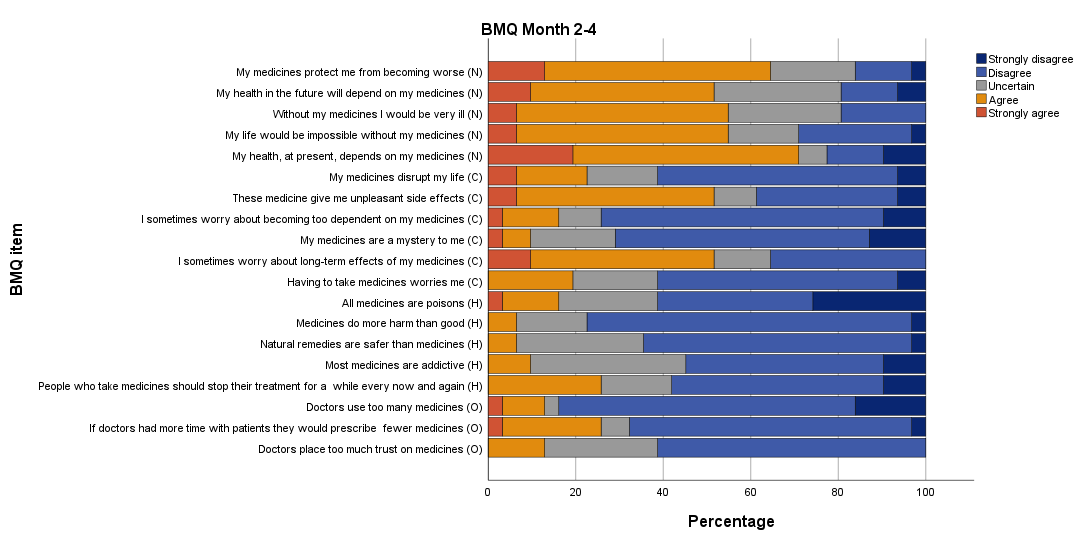


S2c.


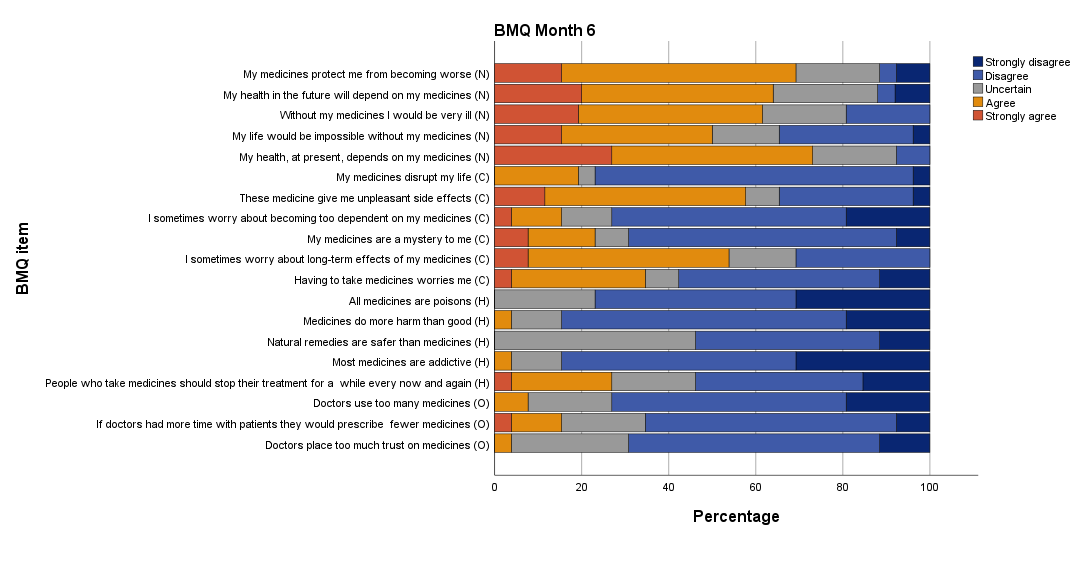


S2d.


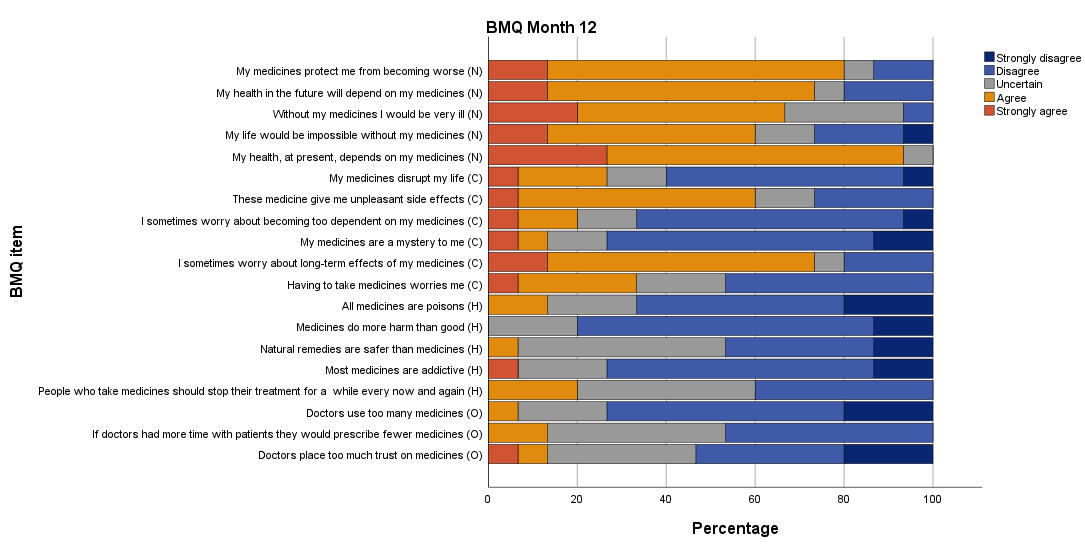


Abbreviations: BMQ, Believes about Medicines Questionnaire; N, Necessity scale; C, Concern scale; H, Harm scale; O, Overuse scale
